# Setting population-size targets for geese causing socio-economic conflicts

**DOI:** 10.1007/s13280-021-01539-5

**Published:** 2021-03-29

**Authors:** Fred A. Johnson, Henning Heldbjerg, Szabolcs Nagy, Jesper Madsen

**Affiliations:** 1 Department of Bioscience – Kalø, Aarhus University, Grenåvej 14, 8410 Rønde, Denmark; 2 Wetlands International, P.O. Box 471, 6700AL Wageningen, The Netherlands

**Keywords:** Conflict, Consensus-convergence model, Geese, Multi-criteria decision analysis, Objectives, Stakeholders

## Abstract

Most European goose populations have increased exponentially, and this has increasingly brought them into conflict with human activities. To manage this conflict, we used multi-criteria decision analysis to help set population targets for a super-abundant population of greylag geese (*Anser anser*). We relied on expert elicitation to assess the consequences of varying goose abundance on nine ecological, economic, and societal objectives. Representatives from national governments and from non-governmental organizations then weighted the objectives based on their perceived relative importance, and we used a consensus-convergence model to reach stakeholder agreement on the tradeoffs among objectives. The preferred population targets for two management units represent about a 20% reduction from current abundances, which from a management perspective would require considerable effort above and beyond current population-control measures. We believe that multi-criteria decision analysis can provide a systematic and transparent framework for building consensus among diverse stakeholders in a wide array of human-wildlife conflicts.

## Introduction

Due to conservation measures, intensification of agriculture, and climate change during the latter half of the 20th century, most goose populations in Europe have increased exponentially (Fox and Madsen 2017). Although geese are highly valued by society for their provisioning and cultural services, expanding goose populations have increasingly brought them into conflict with other human activities (Buij et al. 2017). The result of exponential growth in European goose populations has not only been increasing conflicts with agricultural interests, but problems associated with air-flight safety, human and animal health, ecosystem impacts, and conflict with other biodiversity objectives (Fox and Madsen 2017). A recent supplement of Ambio (Special Issue 2/2017) is testament to the high political profile of goose-human conflicts in the Northern Hemisphere.

To address these concerns, the European Goose Management Platform (EGMP) was established in 2016 under the auspices of the African-Eurasian Waterbird Agreement (https://egmp.aewa.info/) to help reduce human-goose conflict and manage exploitation in a sustainable manner. In the years since, much discussion in the EGMP has revolved around the need to understand and manage the tradeoffs inherent in meeting a diverse set of societal objectives for goose conservation and management. In particular, there has been a great deal of debate about setting abundance targets for goose populations that are subject to recreational hunting and/or cause conflicts with human activities. In some cases, however, the very notion of managing the size of goose populations has been controversial, particularly when increased goose abundance is apparently driven largely by agricultural (i.e., anthropomorphic) changes to the landscape (Fox and Abraham 2017). Nonetheless, the EGMP successfully negotiated flyway-wide abundance targets for pink-footed geese (*Anser brachyrhynchus*) (Williams and Madsen 2013; Madsen et al. 2017) and taiga bean geese (*Anser fabalis fabalis*) (Marjakangas et al. 2015) with limited controversy. However, debate about goose population targets has increased as discussion has turned to species with larger ranges, greater complexity with regard to protection status, and larger adverse societal impacts than those from pink-footed and taiga bean geese.

In light of the controversies surrounding human-goose conflicts, we sought an approach for setting population targets in a fair and transparent manner and, critically, in a way that does not conflate science and values (Pielke 2007). Like all decisions, setting population targets involves both predictions and value judgements. In our case, science is necessary to predict the consequences of alternative population targets relative to various socio-ecological objectives, and value judgements are needed to first define those objectives and then to decide acceptable tradeoffs among them. Multi-criteria decision analysis (MCDA) (Esmail and Geneletti 2018) can be a valuable tool in these contexts. MCDA is a systematic process for predicting the consequences of alternative choices and then using the relative importance of a set of objectives to identify the most preferred alternatives. Widely used in natural resource management, MCDA combines scientific information with value-based objectives to identify a preferred decision alternative (Huang et al. 2011). Examples of application in environmental management are diverse, including water quality management, forest management and restoration, conservation prioritization and planning, protected area planning and management, and resolution of conservation conflicts (Kiker et al. 2005; Huang et al. 2011; Davies et al. 2013; Esmail and Geneletti 2018).

Herein, we demonstrate the utility of MCDA for setting population targets for the NW/SW European population of greylag geese (*Anser anser*), which has increased from about 30 000 in the 1960s to around 1 000 000 today (Powolny et al. 2018). The range of the NW/SW European population of greylag geese includes Norway, Sweden, Finland, Denmark, Germany, Netherlands, Belgium, France, Spain, and Portugal (hereafter collectively referred to as Range States). The EGMP intends to manage the NW/SW population of greylag geese based on two breeding management units (MU):

(1) MU 1 (migratory)

Breeding: Norway, Sweden, Denmark, and Finland

Migratory stopovers: Denmark, Germany, and France

Wintering: Netherlands, Belgium, Denmark, Sweden, Spain, France, and Portugal

(2) MU 2 (sedentary)

Breeding: Germany, Netherlands, Belgium, and France

Wintering: Germany, Netherlands, Belgium, and France

Neither the EGMP nor the EU Birds Directive has a formal procedure for setting targets for huntable species causing management concerns, relying thus far on ad hoc negotiations and consensus-building among stakeholders (Madsen et al. 2017). With the aim of creating a more transparent and replicable process, the EGMP International Working Group in 2018 approved MCDA as a framework for deliberations concerning the setting of management targets for this population. The idea for greylag geese was first to consider fundamental management objectives described in the International Single Species Management Plan (ISSMP) (Powolny et al. 2018) and then use the best available information to predict the consequences of varying levels of goose abundance for each of those objectives. The best choice of a target for abundance is the one that maximizes the weighted sum of consequences across objectives, using objective weights provided by decision makers (Hammond et al. 1999). MCDA explicitly recognizes multiple objectives and inherent tradeoffs and relies on decision makers to determine the relative importance of various management objectives.

Phase I of the MCDA involved identification of the fundamental management objectives of the ISSMP and an assessment of the potential consequences of varying levels of greylag goose abundance. Ideally, the potential consequences of various population sizes are based on empirical models. Although population models for greylag geese are in development, they are not yet ready nor will they be sufficient to address all management objectives. Thus, we relied on expert opinion, which is widely used in the absence of empirical information and can be a valuable tool for decision-making if proper protocols are followed (Morgan 2014).

The expert elicitation was followed by phase II of the MCDA, in which members and permanent observers of the EGMP were asked to assign weights to the management objectives, reflecting their perceived importance of each objective. National Government Representatives (NGRs) and permanent observers of the EGMP participated in this exercise. Participants used a technique known as swing-weighting (Gregory et al. 2012) to identify weights, using the results of the expert elicitation described above. Swing-weighting is an exercise in which decision makers are asked to rank the perceived importance of multiple objectives and to identify acceptable tradeoffs among them.

We describe the methods used in each phase of the MCDA, provide the results of those two phases, and describe and discuss the results of the MCDA in terms of potential population targets. We go on to describe how the application of MCDA could be used effectively to help address a wider array of wildlife-human conflicts.

## Materials and methods

It is important to recognize that a population target differs from a Favourable Reference Population (FRP), which is legally required and defined as the minimum abundance required to sustain an ecologically functional population according to the EU Habitats Directive (Trouwborst et al. 2017). Rather, a population target is to be set above the FRP to help address economic, cultural, recreational, or other societal considerations. We emphasize that, at the time of this research, FRPs had not yet been established by the EGMP for greylag geese; therefore, the objective of maintaining the population above that value was not explicitly considered in the MCDA. Ultimately, any candidate targets that fall below the FRP will be dropped from consideration.

We relied on the ISSMP for specification of other fundamental management objectives. In some cases, we attempted to provide more specificity to the objectives so that it was clear to experts exactly what consequences were being elicited (Table 1). Objectives were described both for breeding (roughly defined as May–July) and wintering (roughly defined as August–April) seasons, recognizing that the effects of abundance might vary between them. An exception was the objective related to hunting opportunity, which is not available during the breeding season. The objectives represent a diverse array of conservation and societal interests.

**Table 1:** Management objectives for the NW/SW European population of Greylag Geese based on the International Single Species Management Plan. Culling, or the so-called derogation kill, is conducted pursuant to Article 9 of the European Union’s Birds Directive to help alleviate human-goose conflicts. Note that at the time of this writing, a Favourable Reference Population had not yet been established by the EGMP; therefore, this objective was not explicitly considered in the MCDA

#	Criterion	Objective
1.	Maximize	Cultural and esthetic values
2.	Minimize	Agricultural damage (real or perceived loss of crop biomass)
3.	Minimize	Government payments to mitigate agricultural damage
4.	Minimize	Direct costs to governments of culling (derogation kill) and scaring geese
5.	Minimize	Indirect costs to governments of public culling (derogation kill)
6.	Minimize	Deleterious impacts to other species resulting from habitat modification
7.	Maximize	Satisfaction with the amount of recreational hunting
8.	Minimize	Amenity fouling and disease transmission (maximize public health)
9.	Minimize	Bird strikes (maximize air safety)
10.	Maximize	Probability that population size remains above the Favourable Reference Population

### Phase I: expert elicitation

For each of the fundamental management objectives, experts were asked to decide which of several candidate relationships they believed best characterized the true relationship between greylag goose abundance and the performance metric provided. Experts were asked to do this separately for the breeding season and for the wintering season in their respective country. We emphasized that it was the general shape of the relationship that was important, rather than the precise values of the functional relationships. Breeding-season relationships were management-unit specific (experts only received a form containing the management unit in which their country was a part), but due to the fact that geese from MU1 and MU2 partly overlap outside the breeding season, the wintering season included greylag goose abundance arising from both management units. The current, approximate country-specific abundances of greylag geese for each season were provided as reference.

**Fig. 1:**
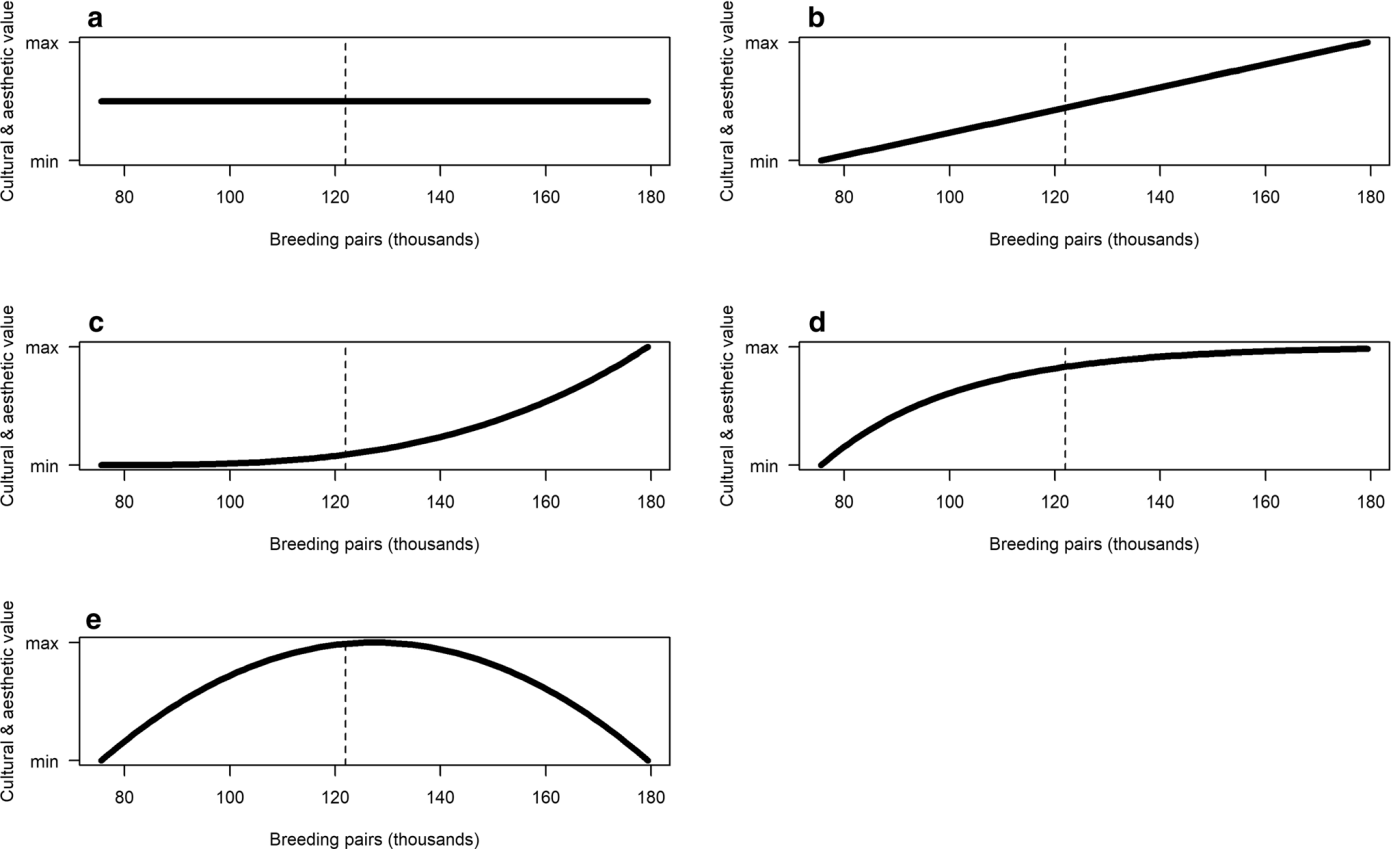
Possible relationships between greylag goose abundance and a management objective (cultural and esthetic values in this case). The vertical, dashed line is current abundance in MU2 as provided in the ISSMP

The candidate relationships provided to experts are shown in Fig. 1, with the scaling of greylag goose abundance depending on the management unit and season. The abscissa thus indicated varying levels of goose abundance and the ordinate represented the consequence for the objective in question. For the candidate relationships (Fig. 1a—flat; b—linear, c—exponential, d—asymptotic, and e—parabolic), the abscissa provided a range of possible abundance values of greylag geese, which included ± 20% of current minimum and maximum values. While this range of values was inherently arbitrary, we believed it was large enough to discern meaningful relationships between abundance and the ability to achieve management objectives. To serve as a benchmark, the approximate, average current values of abundance were shown as vertical dashed lines on the graphs. Breeding-season abundance was in number of breeding pairs, whereas the wintering population was absolute number of individuals (both in thousands). The ordinate represented a relative score corresponding to varying levels of greylag goose abundance and for computational purposes, we allowed this score to range from zero to one. Relationship A (Fig. 1a) posits no relationship between goose abundance and the objective. This might be the case, for example, where impacts occur at a very local level and any relationship with goose abundance may be largely absent at the country level. The parabolic relationship for cultural and esthetic values (Fig. 1e) was available only for this objective because we reasoned that the relationships with other objectives should be monotonically increasing (i.e., never decreasing).

Once experts decided which relationships best characterized the true relationship in their country, they were asked to allocate 100 points among them, reflective of the relative confidence they had in the hypothesized relationships. For example, for cultural and esthetic values an expert may have decided that the relationship was most likely linear (Fig. 1b), but they also believed it might be asymptotic (Fig. 1d). Thus, they might have placed 75 points on the linear relationship and 25 points on the asymptotic one. Experts were instructed not to feel compelled to respond to an objective or season if they did not feel qualified to do so, or if it was not applicable to their country (e.g., recreational hunting is not permitted in The Netherlands, agricultural damage payments are not made in Denmark).

We strongly emphasized to experts that their responses were intended to represent the best available information (i.e., empirical information or expert opinion) and that they should be as objective as possible. The expert elicitation was analogous to a modeling exercise and thus it would have been inappropriate to impart personal values or institutional agendas. Value-based judgements indicating the relative importance of the management objectives are the purview of decision makers and were assessed in phase II of the MCDA.

The EGMP Data Centre (https://egmp.aewa.info/data-centre) identified experts who were known for their scientific work on goose ecology and management in the Range States of the NW/SW European population of the greylag goose. The experts were scientists (rather than decision makers) who work with aspects of human–goose interactions and ecosystem services, including exploitation. The Data Centre contacted at least three experts in each participating Range State, and received responses from the following number of goose experts: Belgium: 2; Denmark: 3; Finland: 2; France: 2; Netherlands: 2; Norway: 3; Spain: 4; and Sweden: 3. We note that Germany is not participating in the implementation of the greylag goose ISSMP, and thus did not participate in the MCDA exercise.

To summarize the relationships for each management unit we used the following protocol:

- Within experts, we averaged the consequence scores for each of the postulated response curves, using the points assigned to each curve as weights.
- Within a country, responses from experts were equally weighted using a simple average because there was no a priori reason to believe some experts were more qualified than others.
- Once breeding-season responses were averaged over experts for each Range State, they were combined for a management unit response using a weighted average, with weights based on the current estimate of breeding pairs in each country (as provided in the ISSMP).
- For wintering-season responses, Range States were also combined using a weighted average, but with weights based on the approximate winter distribution of geese among Range States as determined by neck collar observations (Bacon et al. 2019) (L. Bacon, pers. commun.).

Specification of candidate population targets is somewhat arbitrary, but the goal was to select a range wide enough to encompass diverse stakeholder interests, and with increments that would reflect realistic management and monitoring capabilities. We chose a range of ± 20% of current minimum and maximum abundances because we could not foresee managers desiring populations much larger or smaller than this, nor did we believe it was within their capability to seek more extreme values, especially in light of the tradeoffs among competing objectives. We specified candidate population targets for the two management units in the following manner (all values in thousands):

MU1:

- Range of the number of breeding pairs reported in ISSMP: 81.6 – 92.0 (mean = 86.8)
- Reported range ± 20%: 65.3–110.4
- Five equally spaced values within the range (rounded): 65, 77, 88, 99, 110
- Specified candidates in increments of 10: 70, 80, 90, 100, 110

MU2:

- Range of the number of breeding pairs reported in ISSMP: 94.5–149.5 (mean = 122.0)
- Reported range ± 20%: 75.6–179.4
- Five equally spaced values within the range (rounded): 76, 102, 128, 153, 179
- Specified candidates in increments of 20: 80, 100, 120, 140, 160

Using the weighted curves described above, we constructed a table depicting the consequences of candidate targets for all nine objectives during both the breeding and wintering seasons. The candidate targets were specified as all possible pairs of the five candidates for each management unit. Thus, there were 25 total candidates, expressing possible targets for the two management units. As before, breeding-season and wintering-season consequences were weighted by the relative abundances of greylag geese in each Range State.

The resulting consequence table provided scores for the 25 candidates on each of the nine objectives for each season. The large number of candidates results from what we believed to be a minimum number of candidates for each management unit (5 each). The large size of the consequence table (18 rows, 25 columns) made it difficult, however, to assess the relative tradeoffs among objectives. Low targets generally scored better on objectives like agricultural damage (objective #2), but worse on objectives like cultural and esthetic values (objective #1) and the level of satisfaction with the amount of recreational hunting (objective # 7). While these sorts of general patterns were apparent, the precise extent of the tradeoffs was difficult to assess because of so many objectives and so many candidate targets. There are at least two ways to simplify a consequence table so that the nature of the tradeoffs is more obvious (Hammond et al. 1999). The first is to determine if there are any irrelevant objectives; i.e. those that do not substantially help a decision maker distinguish among the candidate targets. The second is to determine if there are any dominated alternatives; i.e., those candidate targets that perform worse or no better than other candidates across all objectives. We used both approaches to simplify the consequence table.

We first inspected the correlation between breeding and wintering-season consequences for each of the nine objectives, reasoning that if there was a high correlation then the consequences for one of the two seasons were largely redundant. We observed the following Pearson correlation coefficients between the breeding and wintering-season consequences for each objective:

1. Cultural and esthetic values: 0.85
2. Agricultural damage (real or perceived loss of crop biomass): 0.93
3. Government payments to mitigate agricultural damage: 0.98
4. Direct costs to government of culling and scaring: 0.96
5. Indirect costs to government of public derogations: 0.91
6. Deleterious impacts to other species resulting from habitat modification: 0.95
7. Satisfaction with amount of recreational hunting opportunity: NA
8. Public health (amenity fouling & disease transmission): 0.93
9. Air safety (number of bird strikes): 0.72

We arbitrarily chose a correlation coefficient of 0.90 as a threshold, and eliminated the wintering-season consequences for any objective that had a coefficient greater than this. While we could have eliminated the breeding-season consequences instead, we chose to retain them because the focus is on establishing breeding-season targets for the two management units. For cultural and esthetic values and for air safety, the correlation coefficients fell below the threshold of 0.9. For cultural and esthetic values, we chose to retain only the wintering-season consequences because they were generally higher (better) than during the breeding season. We believe this is a logical outcome because geese are concentrated in flocks during the winter and the subject of considerable bird-watching. For air safety (bird strikes), the consequences were also generally higher (worse) during the winter season, again perhaps due to large concentrations of geese. For both objectives, we therefore retained consequences only for the wintering period. For recreational hunting opportunity, we also only used the wintering-season consequences because there is no recreational hunting during the breeding season.

Once we had reduced the consequence table to nine rows, one for each objective, we focused on those objectives related to government costs (objectives #3–5). Because both direct and indirect costs are on the same scale (0–1), we summed them for a total cost. In the expert elicitation, we distinguished among different types of costs because of the possibility that the relationships with greylag goose abundance might differ. However, once those different costs were tabulated for each of the candidate targets, it was possible to simply sum them for a total cost to government. The resulting consequence table now had seven objectives to use in evaluating the 25 candidate targets.

We next turned to identifying any dominated candidate targets. The following candidates did worse or no better than other candidates; i.e. they were “dominated” by other alternatives and thus could be eliminated from consideration (Hammond et al. 1999). The dominated alternatives were (values are in thousands of breeding pairs for MU1/MU2, respectively): 90/80, 90/100, 90/120, 100/80, 100/100, 100/120, 100/140, 110/80, 110/100, 110/120, 110/140. The result was a greatly simplified consequence table consisting of seven objectives and 14 candidate targets. This reduced consequence table was provided to members of the EGMP International Working Group in order to elicit the relative importance of each of the management objectives.

### Phase II: weighting of management objectives

When a decision maker has more than just a few objectives, swing-weighting is one of the easiest methods for determining their relative importance (Gregory et al. 2012). Swing-weighting involves a thought experiment where the participant is first asked to imagine a baseline alternative that has the worst consequences across all objectives. Then the participant is asked to identify their most important objective and to swing its (and only its) consequence from its worst value to its best to develop a hypothetical alternative. That alternative is given a rank of 1 (the best). The participant repeats the process swinging one (and only one) consequence from its worst to its best, and ranks those hypothetical alternatives from the second best (2) to the worst (7, in this case). Then the participant assigns 100 points to the hypothetical alternative ranked number 1. They then assign points to the remaining hypothetical alternatives in accordance with how important they are relative to the top ranked one. Finally, the point values are normalized to provide a relative weight for each of the objectives.

Once objective weights were solicited, they were used to identify a preferred alternative (a set of management-unit population targets in this case). First, all consequence scores from the expert elicitation were normalized to the interval 0–1 (with 0 being the worst outcome and 1 being the best) for each objective. Then for each alternative, a weighted sum of the (normalized) consequence scores was calculated, using the objective weights established in the swing-weighting exercise. Because objective weights varied among members of the EGMP, we used the consensus-convergence model to identify a set of consensus weights (Regan et al. 2006). This method avoids many of the pitfalls of ad hoc methods of negotiation and consensus-building because it is inclusive of all group members, is blind to dominant personalities within the group, and is immune to the influence of powerful special interests. The consensus-convergence model has its foundations in the philosophy of negotiation, and the method is both transparent and repeatable. Basically, the method relies on the correlations in responses among participants. Higher correlations result in more weight on those participants. In other words, participants with more similar objective weights have more influence on the overall average. Extreme views (e.g., almost all of the weight on any one objective) have less influence on the overall average. By agreeing to the application of this method for creating consensus weights, all stakeholders were agreeing to compromise their values to some extent by explicitly recognizing the different values held by others in the group (which, of course, is the basis of any negotiated settlement).

We received objective weights from the national governments of Belgium, Denmark, Finland, France, the Netherlands, Norway, and Sweden, and from the following EGMP permanent observers: The International Council for Game and Wildlife Conservation, the Committee of Professional Agricultural Organizations-General Confederation of Agricultural Cooperatives, BirdLife International, the European Federation for Hunting and Conservation, the European Institute for the Management of Wild Birds and their Habitats, Wetlands International, and the Wildfowl and Wetlands Trust.

## Results

**Fig. 2:**
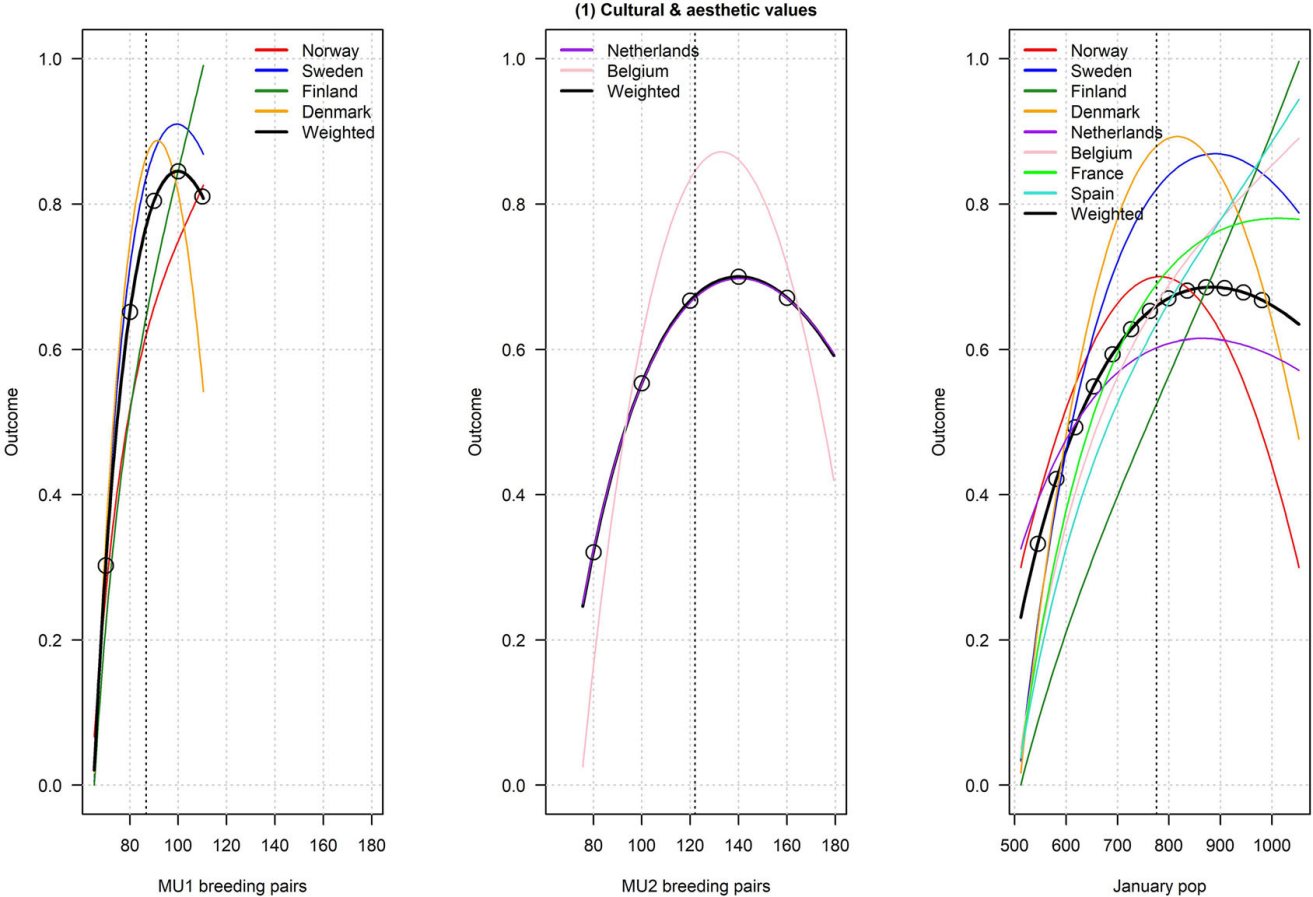
The relationships between greylag goose abundance and cultural and esthetic values as judged by goose experts in the Range States. The circles located on the weighted-average (black) curves for the breeding season depict candidate values for target population sizes for the two management units. The circles on the weighted-average (black) curve for the wintering season depict approximate wintering abundances arising from all possible combinations of the breeding-season candidate targets. Population sizes are in thousands

**Fig. 3:**
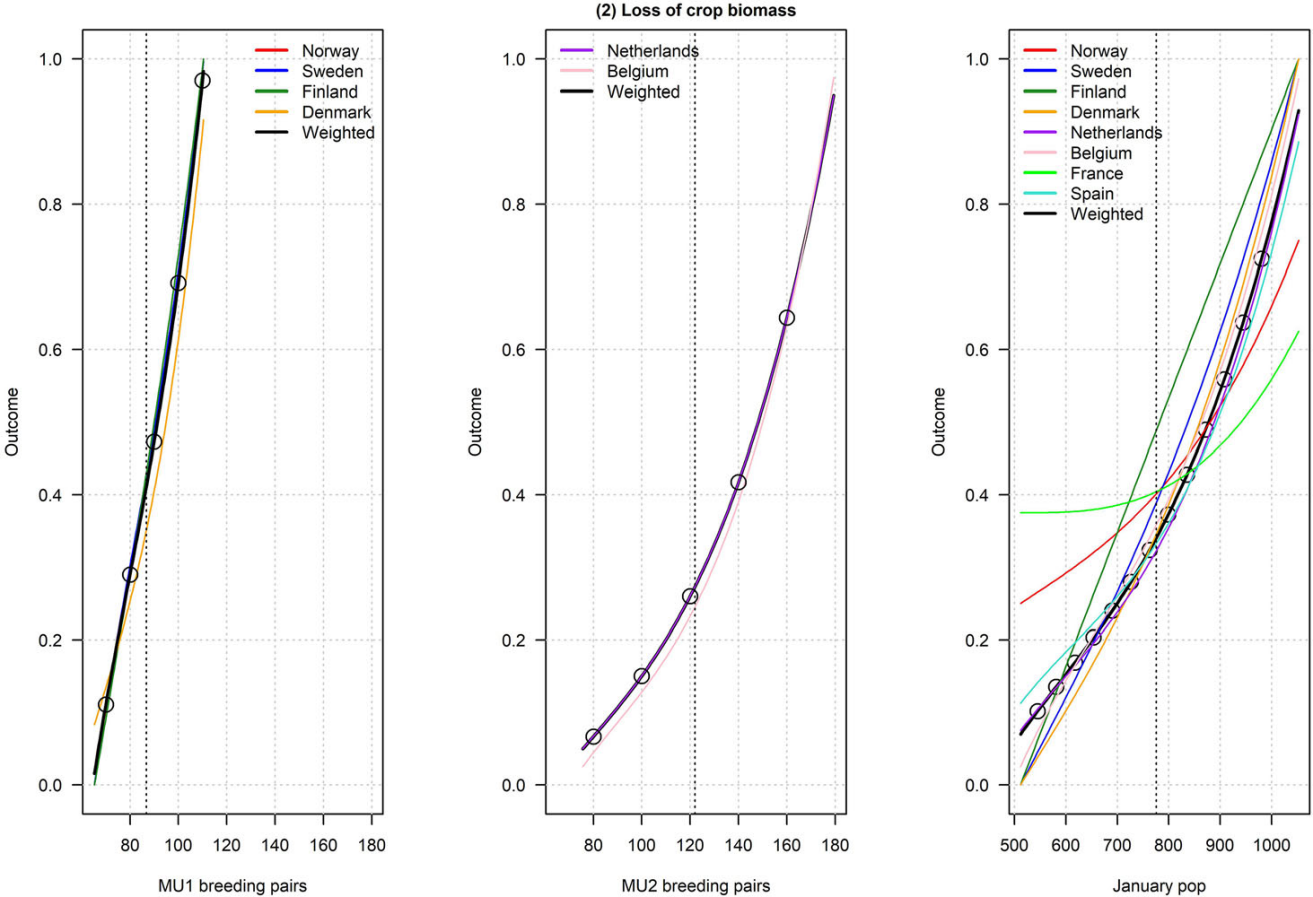
The relationships between greylag goose abundance and loss of crop biomass as judged by goose experts in the Range States. The circles located on the weighted-average (black) curves for the breeding season depict candidate values for target population sizes for the two management units. The circles on the weighted-average (black) curve for the wintering season depict approximate wintering abundances arising from all possible combinations of the breeding-season candidate targets. Population sizes are in thousands

**Fig. 4:**
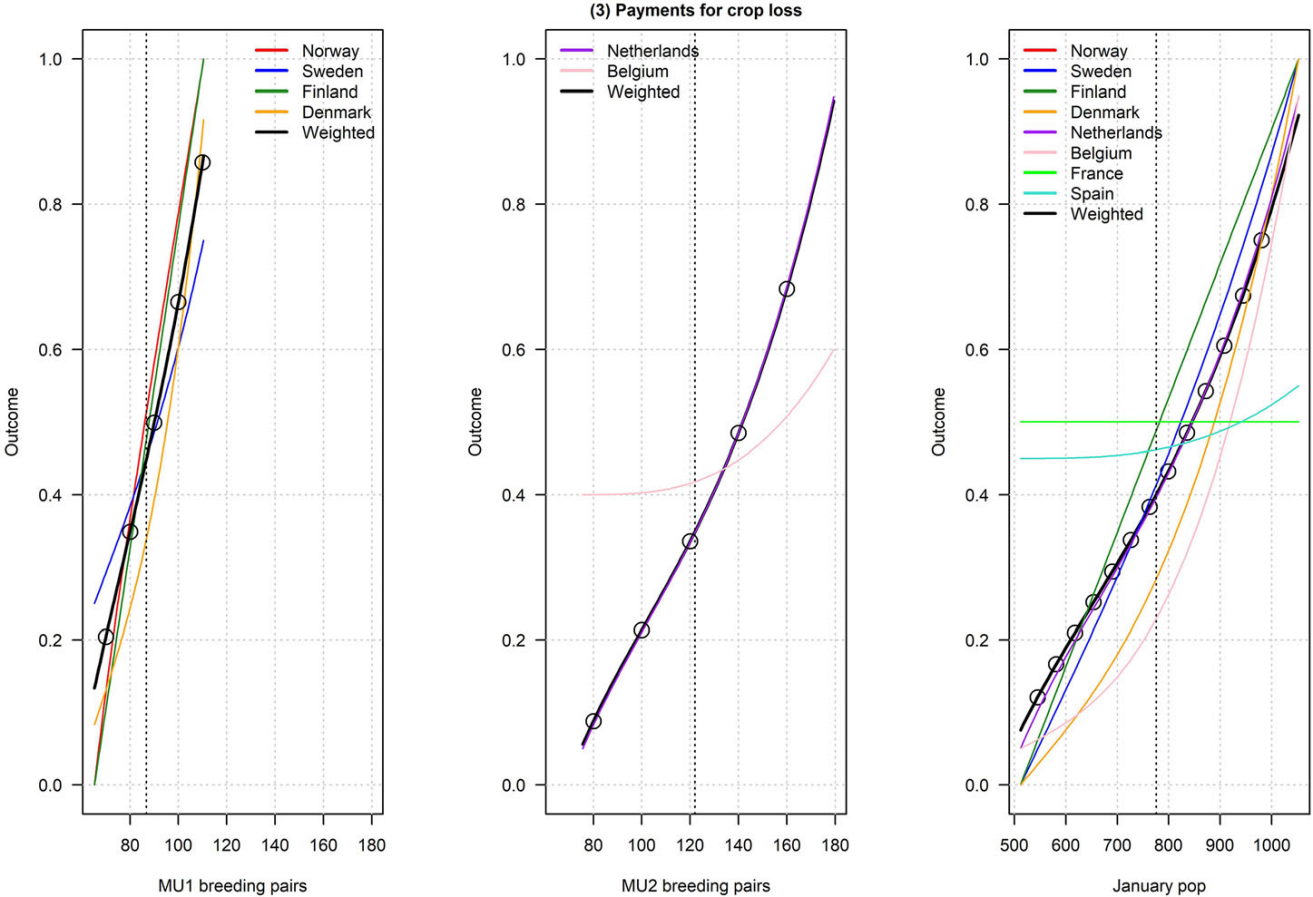
The relationships between greylag goose abundance and government payments to mitigate agricultural damage as judged by goose experts in the Range States. The circles located on the weighted-average (black) curves for the breeding season depict candidate values for target population sizes for the two management units. The circles on the weighted-average (black) curve for the wintering season depict approximate wintering abundances arising from all possible combinations of the breeding-season candidate targets. Population sizes are in thousands

**Fig. 5:**
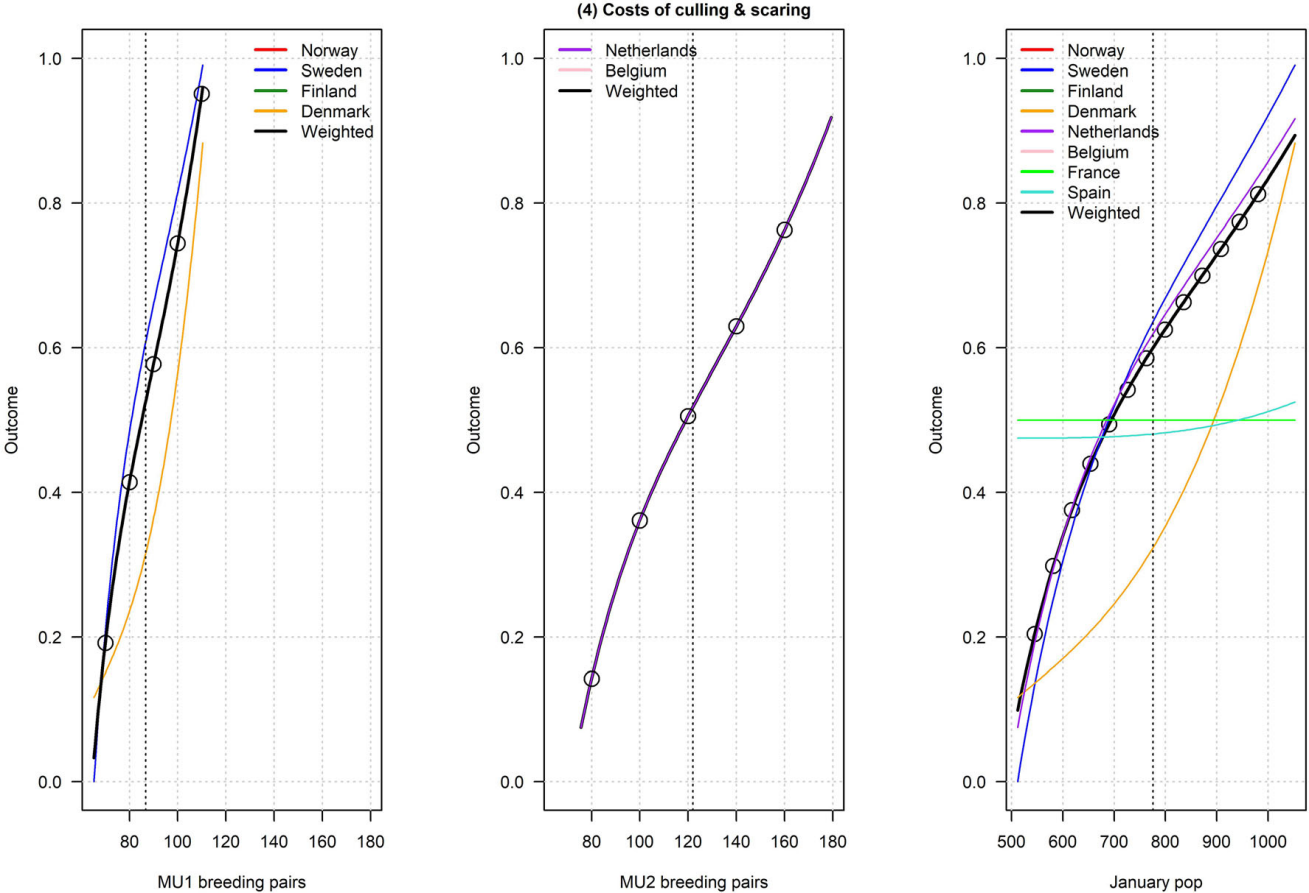
The relationships between greylag goose abundance and direct costs to governments of culling and scaring geese as judged by goose experts in the Range States. The circles located on the weighted-average (black) curves for the breeding season depict candidate values for target population sizes for the two management units. The circles on the weighted-average (black) curve for the wintering season depict approximate wintering abundances arising from all possible combinations of the breeding-season candidate targets. Population sizes are in thousands

**Fig. 6:**
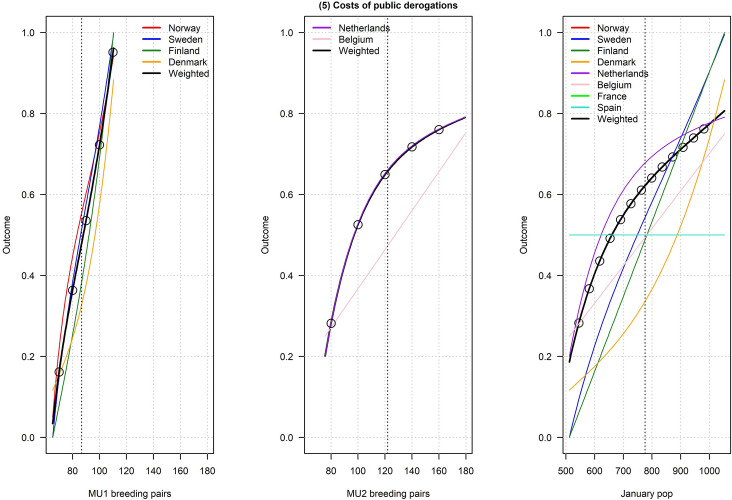
The relationships between greylag goose abundance and indirect costs of public derogations as judged by goose experts in the Range States. The circles located on the weighted-average (black) curves for the breeding season depict candidate values for target population sizes for the two management units. The circles on the weighted-average (black) curve for the wintering season depict approximate wintering abundances arising from all possible combinations of the breeding-season candidate targets. Population sizes are in thousands

**Fig. 7:**
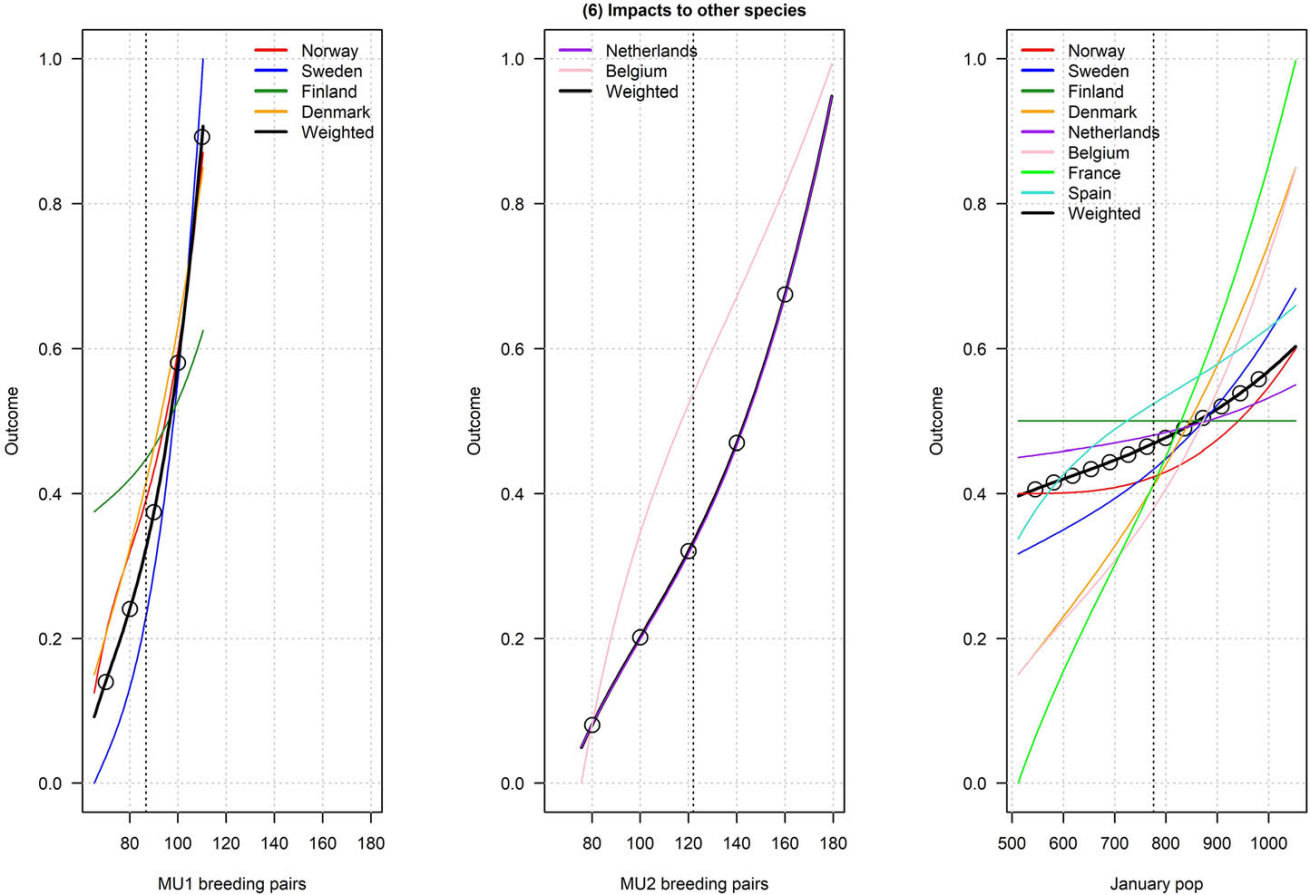
The relationships between greylag goose abundance and deletarious impacts to other species as judged by goose experts in the Range States. The circles located on the weighted-average (black) curves for the breeding season depict candidate values for target population sizes for the two management units. The circles on the weighted-average (black) curve for the wintering season depict approximate wintering abundances arising from all possible combinations of the breeding-season candidate targets. Population sizes are in thousands

**Fig. 8:**
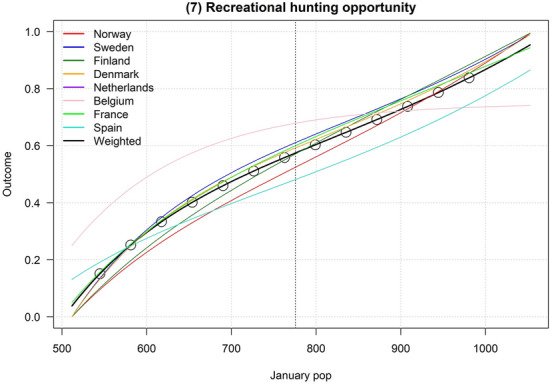
The relationship between greylag goose abundance and satisfaction with the amount of recreational hunting as judged by goose experts in the Range States. The circles on the weighted-average (black) depict approximate wintering abundances arising from all possible combinations of the breeding-season candidate targets. Population sizes are in thousands

**Fig. 9:**
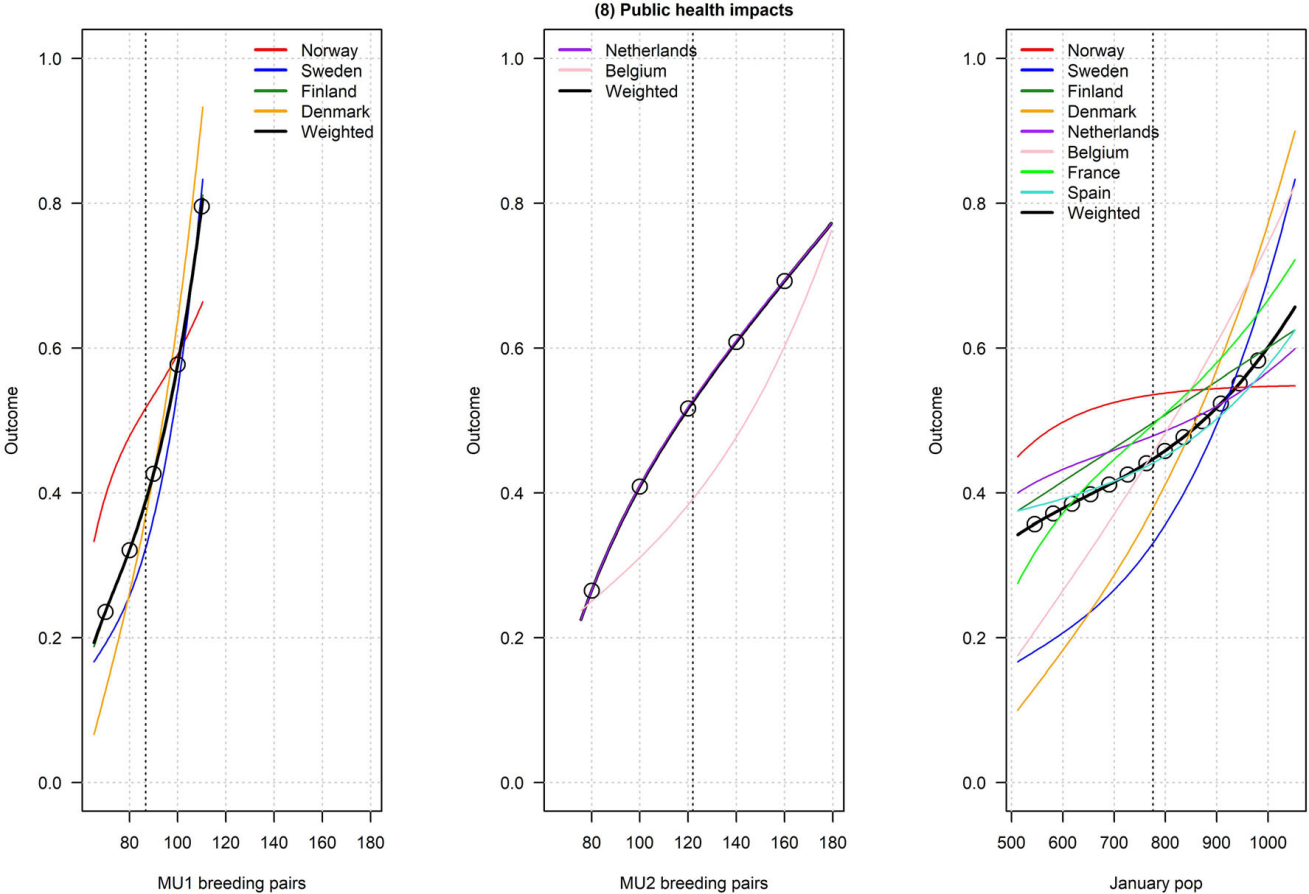
The relationships between greylag goose abundance and public health (amenity fouling and disease transmission) as judged by goose experts in the Range States. The circles located on the weighted-average (black) curves for the breeding season depict candidate values for target population sizes for the two management units. The circles on the weighted-average (black) curve for the wintering season depict approximate wintering abundances arising from all possible combinations of the breeding-season candidate targets. Population sizes are in thousands

**Fig. 10:**
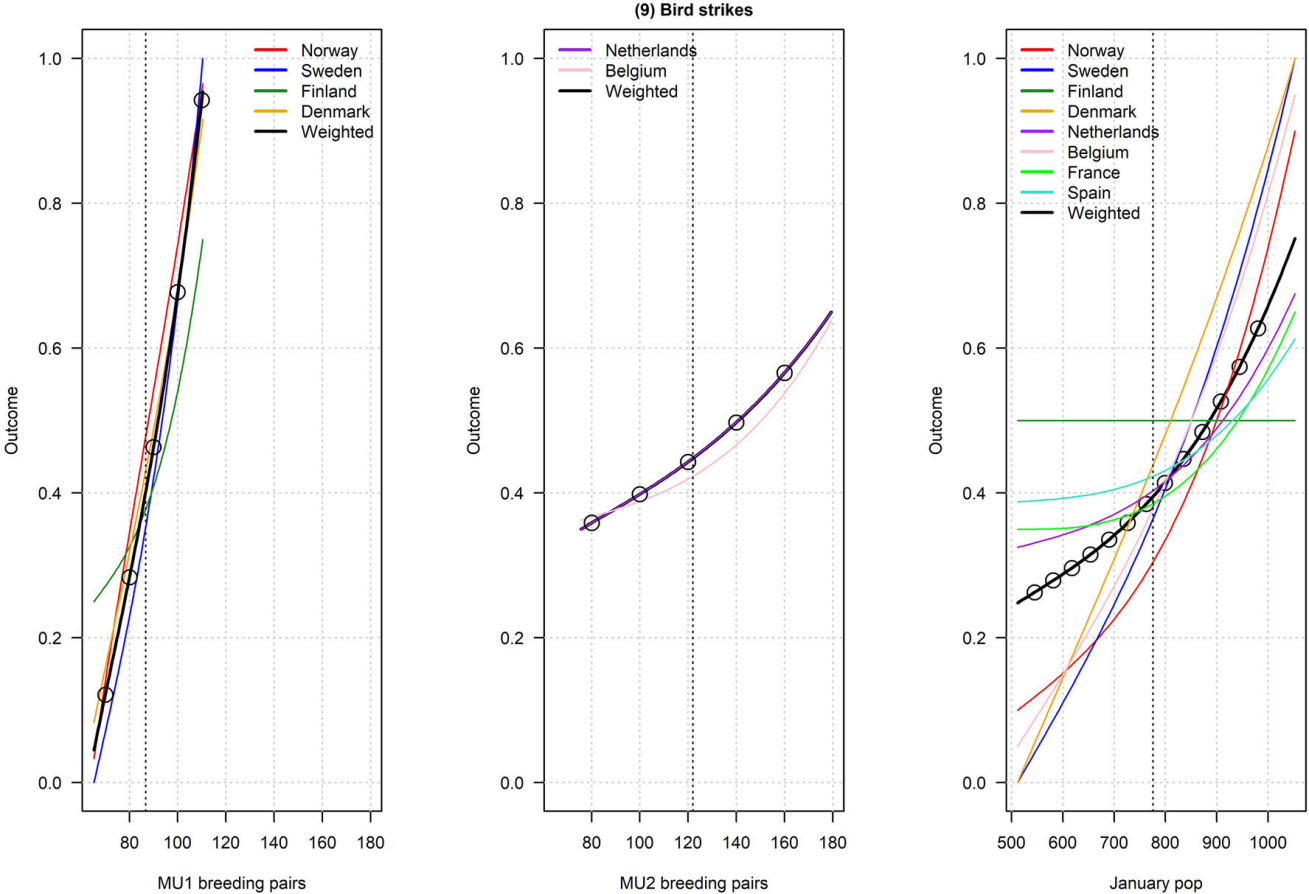
The relationships between greylag goose abundance and air saftey (number of bird strikes) as judged by goose experts in the Range States. The circles located on the weighted-average (black) curves for the breeding season depict candidate values for target population sizes for the two management units. The circles on the weighted-average (black) curve for the wintering season depict approximate wintering abundances arising from all possible combinations of the breeding-season candidate targets. Population sizes are in thousands

Figures 2, 3, 4, 5, 6, 7, 8, 9, and 10 depict responses elicited from experts concerning the consequences of varying goose abundance during breeding and wintering seasons, along with the weighted averages as described in the Methods. Across all objectives, there tended to be more agreement among goose experts in the shapes of the relationships with greylag goose abundance during the breeding season than during the winter. When weighted by country-specific abundance, most relationships were nearly linear, although the slopes of the curves varied among objectives. In particular, the curves were nearly flat for habitat impacts (objective #6) and public health (objective #8) during the winter, suggesting that greylag goose abundance had little influence on those objectives during the wintering period. For cultural and esthetic values (objective #1), the weighted curves were parabolic, reflecting the view that maximization of this objective occurs in the mid-range of greylag goose abundance.

Table 2 depicts the consequence table that was provided to the EGMP national representatives and permanent observers for assigning weights to the management objectives. Note that the goal is to minimize the consequence scores for all objectives except cultural and esthetic values (objective #1) and recreational hunting (objective #7), for which the goal is maximization. As specified in the ISSMP, the current estimated abundance of breeding pairs is approximately 90 000 and 120 000 in MU1 and MU2, respectively. The tradeoffs between low and high goose abundance are readily apparent, suggesting that a compromise is necessary to establish population targets.

**Table 2:** Consequence scores associated with candidate population targets for two management units of Greylag Geese. Management objectives are to maximize cultural and esthetic values, minimize crop damage, minimize management costs to governments, minimize deleterious impacts to habitats, maximize satisfaction with the level of recreational hunting, minimize amenity fouling and disease transmission, and minimize bird strikes to aircraft

Candidate	1	2	3	4	5	6	7	8	9	10	14	15	20	25
MU1 breeding pairs	70	70	70	70	70	80	80	80	80	80	90	90	100	110
MU2 breeding pairs	80	100	120	140	160	80	100	120	140	160	140	160	160	160
Winter individuals	545	617	690	763	835	581	654	726	799	872	835	908	944	981
Objective:														
1. Cultural & esthetic	0.33	0.49	0.59	0.65	0.68	0.42	0.55	0.63	0.67	0.69	0.68	0.68	0.68	0.67
2. Crop damage	0.09	0.13	0.20	0.29	0.42	0.16	0.21	0.27	0.37	0.49	0.44	0.57	0.66	0.78
3-5. Costs	1.13	1.80	2.34	2.86	3.49	1.65	2.29	2.80	3.31	3.93	3.73	4.33	4.81	5.42
6. Habitat impact	0.10	0.18	0.25	0.33	0.46	0.15	0.22	0.29	0.37	0.50	0.43	0.55	0.64	0.77
7. Recreational hunting	0.15	0.33	0.46	0.56	0.65	0.25	0.40	0.51	0.60	0.69	0.65	0.74	0.79	0.84
8. Amenity fouling	0.26	0.34	0.40	0.46	0.50	0.29	0.37	0.44	0.49	0.54	0.54	0.58	0.64	0.74
9. Bird strikes	0.26	0.30	0.34	0.38	0.45	0.28	0.32	0.36	0.41	0.48	0.45	0.53	0.57	0.63

**Fig. 11:**
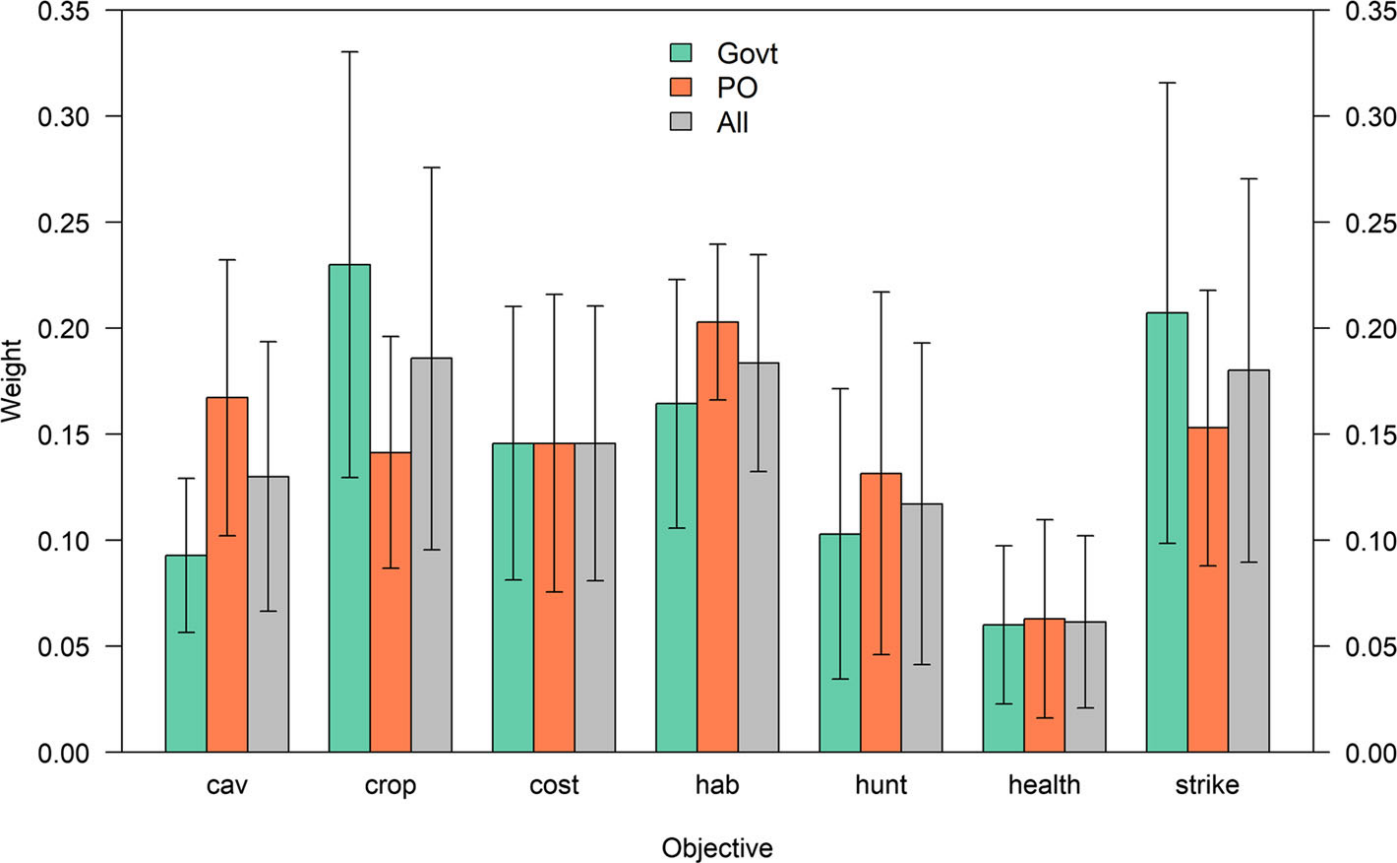
Means and standard errors of the weights assigned to greylag goose management objectives by national governments (Govt), by EGMP permanent observers (PO), and by all respondents. Management objectives are to maximize cultural and esthetic values (cav), minimize agricultural damage (crop), minimize management costs to governments (cost), minimize deleterious impacts to habitats (hab), maximize satisfaction with the level of recreational hunting (hunt), minimize amenity fouling and disease transmission (amen), and minimize bird strikes to aircraft (strike)

Based on responses to the swing-weighting exercise, management objectives to minimize crop damage, adverse habitat impacts, and bird strikes received the highest weights (Fig. 11). There were some minor differences in weights expressed by national governments and those by observers, especially in terms of cultural and esthetic values, crop damage, and bird strikes.

**Fig. 12:**
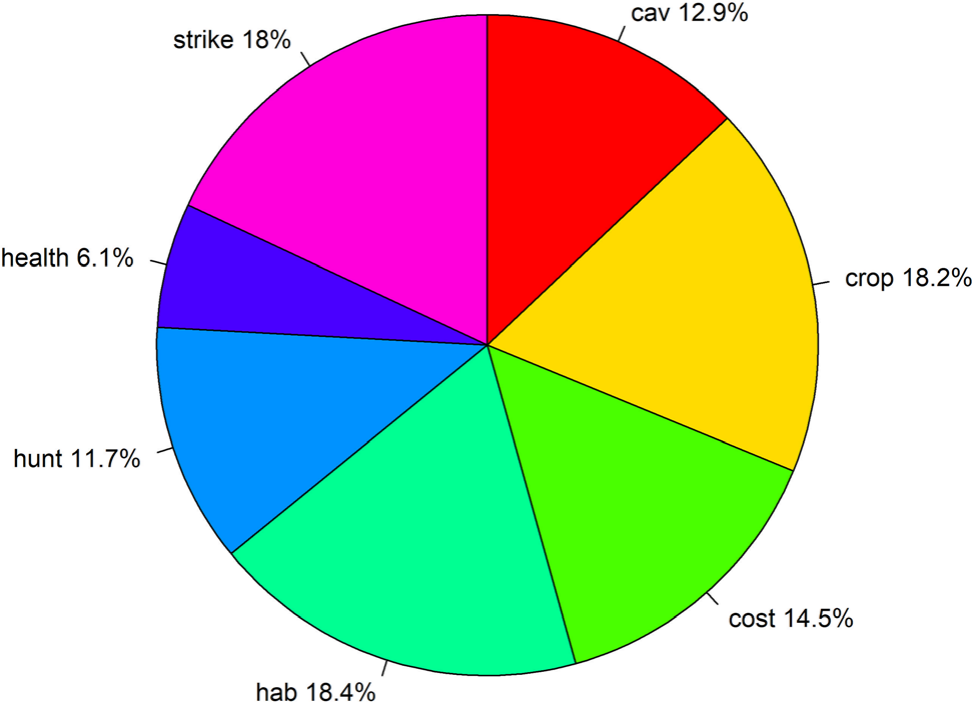
Consensus-convergence weights for greylag goose management objectives derived from EGMIWG respondents. Management objectives are to maximize cultural and esthetic values (cav), minimize agricultural damage (crop), minimize management costs to governments (cost), minimize deleterious impacts to habitats (hab), maximize satisfaction with the level of recreational hunting (hunt), minimize amenity fouling and disease transmission (amen), and minimize bird strikes to aircraft (strike)

**Fig. 13:**
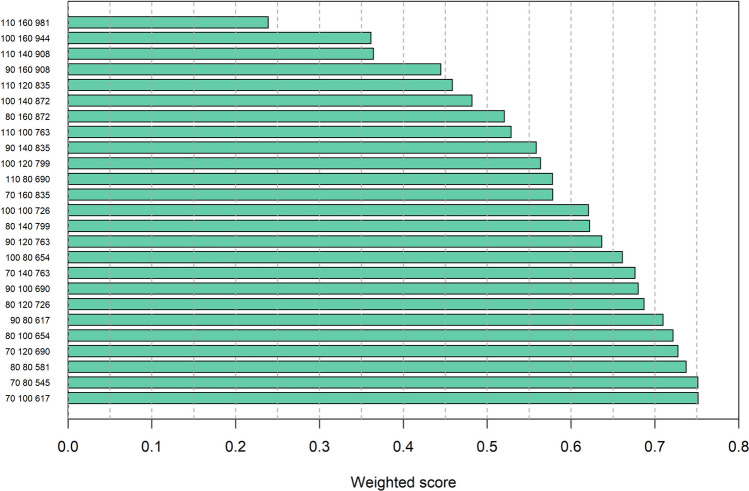
Scores for candidate population targets for greylag geese, weighted by the consensus-convergence weights on management objectives. On the ordinate are first the breeding-pair targets for management units MU1 and MU2, respectively, followed by the approximate number of wintering individuals (all values in thousands). Higher scores indicate higher preference

Using all swing-weighting responses, consensus-convergence weights were highest for habitat impacts, agricultural damage, and bird strikes, intermediate for government costs, cultural and esthetic values, and recreational hunting, and lowest for amenity fouling and disease transmission (i.e., public health) (Fig. 12). Accordingly, the highest scoring candidates tended to be those with the lowest breeding and wintering abundances (Fig. 13). Based on the entire MCDA analysis, the preferred targets for units MU1 and MU2 were 70 000 and 100 000 breeding pairs, respectively (weighted score = 0.7514). However, targets of 70 000 and 80 000 breeding pairs for units MU1 and MU2, respectively, had nearly an identical score (weighted score = 0.7513) to the most preferred candidate. The approximate wintering population size associated with the most preferred candidate is 617 000, compared to 545 000 for the second-most preferred candidate.

## Discussion

To our knowledge, this is the first time that multi-criteria decision analysis has been used to help set population targets for migratory birds. Accordingly, there have been a number of lessons learned. First and foremost, the lack of empirical models to predict the consequences of candidate targets relative to management objectives is an important limitation. Although expert opinion can be a valuable adjunct to empirical data, it is no substitute for direct monitoring of consequences in relation to varying levels of goose abundance. Nonetheless, our elicitation of consequences drew on the expertise of 21 goose specialists in Europe, with a minimum of two experts responding per Range State. The shapes of the relationships between objective consequences and goose abundance were remarkably similar among Range States, particularly during the breeding period, reflecting a high degree of consensus among experts.

Other shortcomings involved the assignment of weights to the management objectives of greylag geese. Ideally, this would involve a fully democratic process, with all members of society having the opportunity to express their opinions. A more practical alternative was to ask the National Governmental Representatives of the Range States and permanent observer organizations of the EGMP International Working Group to best represent the perspectives of their respective stakeholders. Nonetheless, the available time for these parties to consult within their organizations was necessarily limited, and participants in the swing-weighting exercise sometimes expressed frustration at the difficulty of properly representing the diverse views of their constituencies. These limitations imply that the swing-weighting exercise is not repeatable in the sense that different objective weights would likely result if the exercise were repeated.

Despite limitations, the MCDA process as conducted was fully transparent and, importantly, clearly separated the application of science (the expert elicitation) from value-based policy decisions (the swing-weighting exercise). Science and policy issues are often conflated in environmental management, especially in controversial issues (Pielke 2007). The MCDA also identified the nature and extent of tradeoffs inherent in complex decisions, and demonstrated that compromise within and among stakeholder groups would be necessary to reach agreement on population targets for greylag geese. In this regard, use of the consensus-convergence model to identify a set of consensus weights avoided many of the pitfalls of ad hoc, face-to-face methods of negotiation and consensus-building. It is inclusive, repeatable, and transparent, and is immune to powerful special interests that can lead to one-sided agreements. It is notable, however, that the consensus-convergence weights differed little from simple averages among all participants in the swing-weighting exercise. This fact demonstrates that even special interests had regard for the interests of other stakeholders.

Based on the MCDA results, there is near universal agreement that lowering the abundance of greylag geese would best meet a broad range of management objectives. For both management units, the preferred targets represent about a 20% reduction from current values of breeding-season abundance, which from a management perspective would require considerable effort above and beyond current population-control measures. Yet maintenance of the population at a lower abundance could result in substantial long-term cost savings to national governments and agricultural interests, and a significant decrease in the potential for aircraft bird strikes. Lower abundance of greylag geese would be accompanied by some sacrifice from those interested in cultural, esthetic, and hunting values, of course, but even observer organizations of the EGMP International Working Group, which tend to be more conservation and recreation oriented, acknowledged the importance of minimizing the adverse societal impacts of large numbers of geese. In no case, however, can a target population size be set lower than the legally mandated Favourable Reference Population (i.e., the acceptable minimum abundance).

Finally, we acknowledge that the MCDA necessarily represents a coarse-grain analysis, in the sense that we relied on expert opinion for objective consequences, we chose candidate population targets somewhat arbitrarily, and we used a representative rather than fully democratic process for weighting objectives. These facts imply that the weighted scores for the candidate population targets are not precise, in that small differences in scores among candidates are likely not meaningful. Moreover, the most preferred candidates all have values of population targets that are near the minimums considered. Thus, in hindsight, we realized it may have been prudent to examine candidate targets lower than the minimums considered. Before this article went to press, however, the EGMP established FRPs of 35 000 and 73 000 for MU1 and MU2, respectively. The FRP for MU2 is only slightly below the preferred population target and, therefore, there is not much opportunity to set a lower target. For MU1, the preferred target is much greater than the FRP, likely due to the higher interest in hunting in that MU. In that case, a population target much lower than the minimum considered may not be desirable. In any case, we emphasize that the MCDA should not be perceived as dictating a preferred candidate (it is a policy decision after all); rather the MCDA narrows the range of candidates that may be worthy of further discussion, particularly if there are considerations not fully captured by the process.

## Conclusion

We found that MCDA was a valuable tool for decision-making in the EGMP in that it represented a formal and systematic process for better understanding the tradeoffs inherent in managing goose populations causing socio-economic conflicts. The success of the process was confirmed in June 2020 when the EGMP International Working Group formally adopted a set of population targets based on the outcome of the MCDA. Although population targets have been set without the benefit of an MCDA for two other European goose populations (pink-footed geese and taiga bean geese), those populations have relatively restricted ranges, and thus target-setting tends to be less controversial than greylag geese, which are much more abundant and with a wider range. Nonetheless, an MCDA is being considered for setting a potentially new target for pink-footed geese as part of the required revision of its International Single Species Management Plan (https://egmp.aewa.info/resources/action-and-management-plans) in 2024.

Although goose-human conflicts garner a great deal of public attention, human-wildlife conflicts are increasing in other conservation settings as well, such as conflicts between fishing interests and increasing abundance of seals (*Phoca vitulina*, *Halichoerus grypus*) (e.g., Olsen et al. 2018) and cormorants (*Phalacrocorax carbo sinensis*) (Marzano et al. 2013), and between large carnivores and livestock interests (Reinhardt et al. 2012; van Eeden et al. 2018). Herein, we have demonstrated how MCDA can be used to synthesize a wide range of societal objectives into a single target for wildlife abundance, which then can be monitored to determine the population’s response to management (and other uncontrolled environmental drivers). Of course, a critical aspect is that the objectives of conservation and management are related in some meaningful way to wildlife abundance. This will often, although not always, be the case (e.g., where local-scale conflicts are unrelated to wildlife abundance at a larger scale).

Perhaps the most difficult aspect of managing wildlife for a diverse set of stakeholder interests involves the value judgements necessary to decide what makes for acceptable tradeoffs among objectives. In this regard, the consensus-convergence model (Regan et al. 2006) is an extremely useful tool for determining a negotiated agreement on objective weights that avoids the pitfalls of ad hoc, face-to-face negotiations. Yet another approach to negotiated tradeoffs is the concept of Pareto optimality (Kennedy et al. 2007), in which special interests can assess the extent to which they have to sacrifice their favored objectives so that other interests can achieve their objectives. Importantly, this approach does not attempt to aggregate objective scores into a single value using weights, which may miss potentially valuable insight into the nature of stakeholder conflict and how best to address the human-wildlife conflict. We stood ready to pursue the approach of Pareto optimality if the MCDA did not produce a consensus population target. Fortunately, Phase I of our MCDA provided all of the information necessary to examine Pareto optimality, but ultimately the effort was unnecessary. Nonetheless, Pareto optimality can be a valuable approach if the consensus-convergence model fails to deliver broadly acceptable objective weights.

Finally, we stress that the consequences of candidate population targets for various societal objectives are best assessed using empirical information. For example, with geese, it should be possible to compare a temporal sequence of goose abundance and the recorded number of aircraft bird strikes at major airports (Thorpe 2016). It should also be possible to compare agricultural damages and goose abundance, possibly at multiple scales (Baveco et al. 2017; McKenzie and Shaw 2017; Montràs-Janer et al. 2019). In many other human-wildlife conflicts, at least some rudimentary measures of wildlife and the degree and extent of the conflict (e.g., loss of livestock to large carnivores) will be available. However, it will likely be the rare occasion when empirical information is available to address all societal objectives. In these cases, expert opinion can be a valuable adjunct to empirical information, assuming that it is collected in a suitable manner (O’Hagan 2019) and there is explicit acknowledgment of the experts’ uncertainty (Clemen and Winkler 1999; Johnson et al. 2017).

To the best of our knowledge, our research is the first use of MCDA for setting an international target for wildlife abundance in Europe, in which consensus among countries with different cultures, policies, and legislations is necessary. We believe that key to our success was a formal, operational framework for coordinated management (i.e., the EGMP). The other cases of human–wildlife conflict mentioned above (seals, cormorants, large carnivores) are also transboundary issues, but there is no international framework for coordinated management. We believe that significant progress on resolving these conflicts will require the establishment of such frameworks to develop a systematic and transparent approach to collaborative decision-making.
